# Adaptation, modification, and psychometric assessment of a Norwegian version of the Basel extent of rationing of nursing care for nursing homes instrument (BERNCA-NH)

**DOI:** 10.1186/s12913-019-4817-3

**Published:** 2019-12-16

**Authors:** Rebecka Maria Norman, Ingeborg Strømseng Sjetne

**Affiliations:** 1 Norwegian Institute of Public Health, PO Box 222 Skøyen, NO-0213 Oslo, Norway; 2 Department of Health Management and Health Economics, University of Oslo, Faculty of Medicine, Institute of Health and Society, PO Box 1130 Blindern, NO-0318 Oslo, Norway; 3 Lovisenberg Diaconal University College, Lovisenberggata 15b, NO-0456 Oslo, Norway

**Keywords:** Psychometrics, Surveys and questionnaires, Validity, Reliability, Factor analysis, Nursing homes, Long-term care, Rationing, Unfinished care, Care workers, BERNCA-NH

## Abstract

**Background:**

To our knowledge, no instrument has been developed and tested for measuring unfinished care in Norwegian nursing home settings. The Basel Extent of Rationing of Nursing Care for Nursing Homes instrument (BERNCA-NH) was developed and validated in Switzerland to measure the extent of implicit rationing of nursing care in nursing homes. The BERNCA-NH comprises a list of nursing care activities in which a care worker reports the frequency to which activities were left unfinished over the last 7 working days as a result of lack of time. The aim of this study was to adapt and modify a Norwegian version of the BERNCA-NH intended for all care workers, and assess the instruments’ psychometric properties in a Norwegian nursing home setting.

**Methods:**

The BERNCA-NH was translated into Norwegian and modified to fit the Norwegian setting with inputs from individual cognitive interviews with informants from the target population. The instrument was then tested in a web-based survey with a final sample of 931 care workers in 162 nursing home units in different parts of Norway. The psychometric evaluation included score distribution, response completeness and confirmatory factor analysis (CFA) of a hypothesised factor structure and evaluation of internal consistency. Hypothesised relation to other variables was assessed through correlations between the subscale scores and three global ratings.

**Results:**

The Norwegian version of BERNCA-NH comprised four subscales labelled: routine care, ‘when required’ care, documentation and psychosocial care. All subscales demonstrated good internal consistency. The CFA supported the four-factor structure with fit statistics indicating a robust model. There were moderate to strong bivariate associations between the BERNCA-NH subscales and the three global ratings. Three items which were not relevant for all care workers were not included in the subscales and treated as single items.

**Conclusions:**

This study found good psychometric properties of the Norwegian version BERNCA-NH, assessed in a sample of care workers in Norwegian nursing homes. The results indicate that the instrument can be used to measure unfinished care in similar settings.

## Background

The population is aging, with increasing need for complex care in the latter part of life [1–3]. Manpower requirements in the long-term care sector are expected to be twice as high, in man-years, by 2060 compared to today [4]. The cost of care is expected to increase while there will also be problems recruiting and retaining a qualified workforce [5]. Against this background, a widening gap between healthcare demands and available resources in the coming years could be anticipated. Likewise, Kitson et al. [6] argue that there is challenge in meeting the fundamental or basic care needs of patients, and a tension in nursing between “tasks and time”, as well as a challenge in maintaining an interpersonal relationship with patients [6]. Due to scarce resources and lack of time, care workers have to prioritise which activity to complete first. The activity can be deemed as necessary but, when faced with a lack of time, a nurse may have to perform the activity later, more quickly, with less quality, or the activity may be left unfinished [7]. Unfinished care adversely affects the quality of care [8] and have serious consequences for patient safety as they may not receive adequate treatment and care [9].

There is a growing international body of evidence concerning unfinished care and its associated factors in hospital settings [10]. Associations have been found between unfinished care and outcomes such as patient satisfaction, nurse-reported medication errors, patient falls, nosocomial infections, pressure ulcers, critical incidents [11, 12], patients’ experience of patient-centred care [13] and in-hospital mortality [14], as well as individual nurse level variables such as job satisfaction [15]. In nursing home settings the evidence is more scarce [16, 17], but studies conducted in the setting have observed that unfinished care is related to quality of care [18], staffing levels, teamwork, safety climate and work stressors [17], patient outcomes [16] and care workers’ health [19].

In a recent review of staffing levels and omission of care, 14 out of 18 included studies found negative associations between nurse staffing levels and levels of unfinished care in hospitals [20]. To surveil the prevalence of unfinished care may provide an early warning sign for identifying units with low staffing levels [21], and is *“a promising indicator of nurse staffing adequacy”* ([20] p. 1475). Furthermore, there is evidence from hospital settings that unfinished care as assessed by nurses could be used as an indicator of the overall quality of care [21, 22].

In nursing home settings, the group of older patients suffers, to a large extent, from cognitive impairments and such patients are more likely to have a limited understanding of their situation, as well as a reduced ability to self-care and to express their own needs [23, 24]. Thus, this group of patients is likely to have complex, basic nursing care needs which, in turn, require more personnel time [25]. Leaving basic nursing care for these patients unfinished may lead to further functional and cognitive decline [26], although the outcomes may not necessarily be immediately observable. There is also evidence that delayed or inappropriate interventions, medication errors, falls and unfinished nursing care are factors that contribute to most of the serious adverse events in nursing homes [27]. Hence, it is crucial to ensure that basic nursing activities are performed because this can determine patient outcomes [28]. Providing basic nursing care to patients who lack the capacity to self-care is a crucial activity and lies at the core of nursing and combines the “physical, psychosocial/relational dimensions of care” [6, 29]. Regulations stipulate the care to be expected in Norwegian nursing homes and include the requirement that basic care needs should be ensured and individualised through patient involvement [30]. Nursing care should be contextual and adapted to the specific situation and patient [31, 32].

It has been argued that care workers tend to focus on clinical and biomedical activities, leaving out basic needs [26, 33, 34] and social care [17] when prioritising, although psychosocial and social care is reported as important for quality of care [35] and quality of life [36] for patients in nursing homes. Moreover, patients in nursing homes tend to find activities meaningful which address psychological and social needs, whereas the care workers tend to believe that activities that retain physical abilities are more important [37].

Given the prevalence and risk associated with unfinished care, a tool to measure the phenomenon will give important and actionable information about bedside care quality [16]. To our knowledge, the Basel Extent of Rationing of Nursing Care for nursing homes (BERNCA-NH) is the only instrument that has been developed and evaluated to measure unfinished care in nursing homes [3]. The BERNCA-NH was developed and validated in Switzerland. The instrument presents an inventory of basic care activities commonly performed in nursing homes and care workers indicate how often each activity was left unfinished over their last seven shifts due to time constraints.

Different terms have been used for unfinished care, for example, “missed care”, “care left undone” and “omitted care”. In the BERNCA research approach “implicit rationing of care” is used. However, different research approaches refer to the same phenomenon and “unfinished care” has been suggested as an umbrella term in a state-of-the-science review [38]. According to Schubert et.al [7], unfinished care occurs during the process of care [39] and can be conceptualized *as “… a three-pronged phenomenon consisting of a problem (resource/time scarcity), a process (clinical decision making to prioritize and ration care), and an outcome (care left undone)”* ([38] p. 1122) and converges with Donabedian’s [40] structure-process-outcome framework [8]. In BERNCA’s rationale, “implicit rationing of care” in acute care settings is defined as *“the withholding of or failure to carry out necessary nursing measures for patients due to a lack of nursing resources (staffing, skill mix, time*)” ([7] p. 417). “Implicit” in this context denotes a prioritisation that is indirect and unintentional [3, 26] as opposed to “explicit”, in which the priorities are set formally, e.g. in policy and budget processes. Implicit rationing is an individual implicit ad-hoc choice to not carry out certain care activities because of constrained resources [7, 17], such as patient-to-nurse ratios [10].

Norwegian nursing homes are financed through general taxation, and they have an average of 42 beds [41]. In 2017, care workers in Norwegian municipal health care (including nursing homes and home health care) comprised approximately 35% registered nurses (RNs) with a bachelor’s degree, 40% practical nurses (PNs) with upper-secondary education and about 25% nurse assistants (NAs) and other personnel [42]. NAs and PNs carry out approximately the same care [43], and there are no regulations concerning skill-mix or minimum staffing levels in Norwegian nursing homes [44]. Norway and Switzerland are similar in being high-income countries with nursing home services providing services to older people with extensive needs [44, 45]. In the RN4CAST study, the prevalence of nursing care tasks left unfinished in hospitals in Norway and Switzerland was similar [10]. This indicates that the phenomenon is known to care workers in both countries, and that a Swiss tool may be useful after translation and adaptation for measuring unfinished care in a Norwegian setting. Optimal usefulness means that the items cover relevant and important topics, and also that the measures are presented in a format that mirrors the working life of the care workers in the Norwegian nursing home setting. This to ensure that the results can be viewed as relevant for discussions in the specific setting.

Thus, the aim of this study was to adapt and modify a Norwegian version of the BERNCA-NH intended to be applicable for all care workers, and assess the psychometric properties in a Norwegian nursing home setting. The following was carried out: 1) translation, cultural adaptation and modifications, and; 2) assessment of psychometric properties.

## Methods

### The original BERNCA-NH instrument

The 19-item Basel Extent of Rationing of Nursing Care for Nursing Homes instrument (BERNCA-NH) [3] is based on the BERNCA instrument that was developed for RNs in acute hospital settings [7, 11]. The target population for the nursing home version includes all categories of care workers. The introduction to the BERNCA-NH states*: “The questions in this part of the questionnaire address care interventions and therapies that are NECESSARY and USUAL but could not be performed or only partly performed because of LACK OF TIME or HIGH WORKLOAD. How often in your last 7 working days did it happen that…”* The introduction is followed by an inventory of basic care activities performed in nursing homes, the items are stated like for example, item 8: *“…you could* ***not*** *have a conversation with a resident or his / her family?”.* The response options are “Never”, “Seldom”, “Sometimes”, or “Often”. The response option “Activity was not necessary” is offered when applicable and one item has the option “Not within my field of responsibility”. The psychometric properties of the BERNCA-NH were evaluated in a sample of 4748 care workers in the German, French and Italian-speaking regions of Switzerland. Using exploratory and confirmatory factor analysis, an almost identical four-factor structure was found in the three language versions. The subscales in the original Swiss BERNCA-NH were as follows: activities of daily living (ADL) (5 items, for example items on skin care and oral hygiene); caring, rehabilitation and monitoring (8 items, for example emotional support, activating or rehabilitating care, and toileting/continence training); documentation (3 items); and social care (3 items, for example scheduled single /group activities and cultural activities). The two sub- scales, social care and documentation, were clearly distinguished, while the two subscales, ADL and caring, and rehabilitation and monitoring, showed some cross-loadings. All subscales had acceptable internal consistency in the three different versions. Based on the findings, the Swiss validation article concluded that future research should revise and specify more representative items for the subscale social care. Further suggestions were to collapse the items about eating and drinking to one, and to add an item about medication administration to the instrument [3].

### Translation and cultural adaptation

Permission to translate and adapt the BERNCA-NH was obtained from the authors. The aim of cultural adaptation was to ensure that the Norwegian version of BERNCA-NH should be applicable for all care worker occupations and measure relevant aspects of unfinished care in a Norwegian setting. The BERNCA-NH items were translated from German into Norwegian and back-translated independently by different translators, fluent in both languages. The translation followed established procedures [46, 47]. The Norwegian version was finalised in a consensus- finding process between the translators.

Two thirds of care workers in nursing homes have completed upper-secondary school education or less and a large group has a foreign mother tongue. We pretested the Norwegian instrument through individual cognitive interviews, with the aim to detect potential problems with the wording or the response formats, and to examine content validity and cultural relevance [48, 49]. We wanted informants from different occupations and with different mother tongue, and used snowball sampling to recruit 14 informants [50]. The informants comprised of nine PNs, two NAs and three RNs, of those five had Norwegian as mother tongue. The informants filled in their answers to the instrument and their comments were collected by a mixture of think-aloud and concurrent verbal probing [48, 49]. The interviews were conducted in two rounds with adaptions of the items between the rounds.

### Psychometric testing

#### Design and setting

We collected data for the psychometric testing in a cross-sectional survey. The respondents were contacted via their workplace and we attempted to invite all Norwegian nursing homes. Finally, 66 nursing homes (16 to 120 beds) agreed to participate, representing 162 units located in different parts of Norway, including urban and rural districts. The individual inclusion criteria were RNs, PNs or NAs, defined as care workers, working a minimum of 50% in direct patient care, day and/or evening shifts.

#### Data collection

A contact person in each nursing home/unit sent us a list of care workers based on the inclusion criteria and included background information. The list included age group (under 40/over 40 years), occupation (NA, PN, or RN), and mother tongue (Nordic/non-Nordic) for each care worker. We then provided named and closed envelopes for each included care worker, which were distributed by the contact person. The envelopes contained a one-page invitation letter with information about the survey, privacy protection and a specific username and password required for participating online. In addition to the BERNCA-NH, the survey contained items on the care environment, patient safety as well as global ratings and demographic information. The data was collected from September to December 2017. Information and reminders were sent to the units’ contact persons by email and regular post four times during the data collection period.

#### Statistical analysis

IBM SPSS Statistics for Windows (version 24, IBM Corporation, Armonk, NY, USA) was used for all analyses, except for confirmatory factor analysis, for which the Lavaan package [51] in R statistics software (version 3.4.1) [52] was used.

#### Response rate

In order to explore potential non-response bias, the background data of the respondent and the non-respondent group were compared using chi-square statistics.

#### Response completeness

The quality of the data was initially evaluated by examining the score distribution and the proportion of non-valid responses for single items, as well as the instrument as a whole. There could be three types of non-valid responses: The first type is “Item missing” in which an answer is omitted completely. The second and third type are the responses: “Not within my field of responsibility” and “Activity was not necessary”, hereby jointly defined as “Not applicable”. Given the subject matter and the heterogeneity of the care worker sample, a relatively large proportion of these responses had to be expected. The extent of the use of these options were assessed to evaluate the relevance of the items for all care worker occupations. In order to complement the examination of the non-valid responses, we tested the “Not applicable” and “Item missing” by the variables occupation and mother tongue, using survey data.

#### Response variability

Response variability was evaluated based on frequencies, mean and standard deviation on the item scores. High scores represent a higher prevalence of unfinished care: range 1 “Never unfinished” to 4 “Often unfinished”. Scale means were transformed linearly, range 0–100.

#### The subscale structure

We checked whether the data was suited to factor analysis [53], including normality assessed by P-P plots [54], and bivariate linearity assessed by scatterplots among pairs of variables with highest skew and kurtosis [55]. Three items representing activities that were evidently not relevant for all the three occupations were not included in the factor analysis. Since the Norwegian items were adapted and differed to some extent from the original BERNCA-NH, the internal structure was first assessed with exploratory factor analysis. We used principal axis factoring (PAF) and an oblique rotation method (Promax) as we assumed the factors to be correlated [53]. The factor analysis was performed using list wise deletion. We did not find empirical support for a factor structure in our data. For the benefit of the discussion and actions intended to follow the survey in the nursing homes we constructed subscales with a more focused scope than the instrument as a whole. The subscales were constructed with input from two other sources. Firstly, both authors were familiar with the organisational setting and the care activities after several years as RNs and first line managers in nursing homes. This knowledge was useful in order to structure the items according to contents and thereby provide measures that would be recognised and have face validity in everyday bedside practice. Secondly, we turned to literature describing different types of nursing situations [31, 56, 57]. The item assignment was done by the authors independently and finalized in a consensus finding process.

In the first subscale we included activities that are typical for stable and well-known situations. The activities are predictable and take place repetitively, and the procedures are often well known to both patients and care workers. For example, skin care or help with eating. Due to the routine character of the activities, delegation or postponement can be an acceptable solution when the circumstances call for prioritisation. In the second subscale we assigned items that represent activities that cannot be postponed but become unfinished unless they are performed promptly. They are commonly occurring in nursing homes but are less predictable than routine activities. The activities cannot be scheduled, for example necessary patient monitoring or assist to the toilet when needed. Items representing activities that tend to the patients’ psychosocial needs were assigned to a third subscale, and finally items about documentation were assigned to a fourth subscale.

#### Internal consistency of the subscales

The internal consistency of the constructed subscales was assessed by item–total correlations (> 0.3 is considered acceptable). Each item’s contribution to the scale’s Cronbach’s α was assessed [53]. Cronbach’s α (> 0.7 is considered acceptable) was used for assessing the internal consistency of each subscale [58].

#### Confirmatory factor analysis

We performed confirmatory factor analyses (CFA) to evaluate whether our model fitted the data, and to compare the fit statistics with alternative models. We used the diagonally weighted least squares estimate which use polychoric correlations and the full weight matrix to compute robust standard errors, and a mean- and variance-adjusted test statistics, appropriate for ordinal data. All non-valid responses were handled by listwise deletion. The factor loading estimates was required to be > 0.35 [59]. The model was assessed with the comparative fit index (CFI) and Tucker-Lewis index (TLI), values > 0.95 indicates a good fit [58, 60]. The cut-off for root mean square error of approximation (RMSEA) is < 0.06 for a good fit. The standardized root mean square residual (SRMR) should be as low as possible < 0.08 indicates a good fit [60].

#### Validity based on relation to other variables

Previous studies in hospital and nursing home settings have found associations between unfinished care and quality of care, job satisfaction and work environment [7, 10, 15, 17, 18]. Therefore, we hypothesised an inverse, moderate to strong correlation (> 0.30) between the BERNCA-NH subscale scores and three global rating questions included in the survey about the following: (1) overall quality of care; (2) overall job satisfaction and (3) if respondent would recommend unit as a workplace. These were all scored on a scale of 1–10, where 10 is the best possible score.

## Results

### Translation and cultural adaption

Supported by suggestions in previous articles about the instrument, we added two items before the translation: change of wound dressings and medication. For the Norwegian version we collapsed the two items on assistance in eating and drinking into one and revised the social care subscale as suggested in the previous Swiss validation article [3].

In our pre-test cognitive interviews, the informants confirmed the importance and relevance of the topics in the original BERNCA-NH, suggesting that the content of the Swiss instrument can be used to measure unfinished care validly in Norwegian nursing homes. However, according to our informants, social activities can rarely be planned in advance, as one never knows if there will be time to do them. Social activities were performed spontaneously when the occasion allowed. Social activities were also largely undertaken by non-care employees. These results are in line with the Swiss validation article [3]. Therefore, the original items (items 17, 18, 19) on social care were modified. Moreover, the term “continence training” was changed as this was an unfamiliar word among the informants (item 9). We changed item 10 from “Activating or rehabilitating care” to “Allow necessary time for patients to perform care themselves when possible, in order to retain functioning”. In addition, we added a new item about providing food (item 4) between regular mealtimes, as this was a topic deemed by our informants to be missing, as well as a highly relevant aspect of care quality. The word “resident” was changed to “patient” in line with the legal definition in Norway [61]. The response format was the same as the original BERNCA-NH but due to our heterogeneous sample, the response option “Not within my field of responsibility” was included for all items to assess the suitability of the items for all care workers. The Norwegian version of the instrument consisted of 20 items.

### Psychometric properties

#### Response rate

When comparing background information about the respondents with the non-respondents (Table 1), significant differences were found in the occupation and mother tongue groups. There were 5.1% NAs among the respondents compared to 12.8% in the group of non- respondents, and 42.1% were RNs among the respondents compared to 29% in the group of non-respondents. Among the respondents 14.6% had non-Nordic mother tongue, compared to 22.9% of the non-respondents.

**Table 1:** Background information about respondent and non-respondent

		Respondents ^a^(*N* = 953)		Non-respondents *(N* = 1615)		Total (*N* = 2568)		^*b*^Difference
		*N*	%	*N*	%	*N*	%	*p*
Age	Over 40 years	635	66.6	1060	65.6	1695	66	0.606
	Under 40 years	318	33.4	555	34.4	873	34	
Occupation	NA	49	5.1	207	12.8	256	10	< 0.001
	PN	503	52.8	940	58.2	1443	56.2	
	RN	401	42.1	468	29	869	33.8	
Mother tongue	Nordic	814	85.4	1245	77.1	2059	80.2	< 0.001
	Non-Nordic	139	14.6	370	22.9	509	19.8	

^a^Night shift workers (*N* = 22) were excluded in analysis

^b^ χ^2^-test

Among the 2568 care workers in the sample, 953 completed the web-based survey tool giving a 37.1% response rate. After excluding care workers working mainly night (*N* = 22), the final sample was comprised of 931 care workers. Sample descriptives are shown in Table 2.

**Table 2:** Sample descriptives *(N* = 931) based on survey data

		*N*	%
Age	Years, M = 45.4 (SD = 11.9)		
Gender	Female	875	95
	Male	46	5
Occupation	NA	47	5
	PN	490	52.6
	RN	394	42.3
Mother tongue	Nordic	782	84.9
	Non-Nordic	139	15.1
Employment (%)	100	360	39
	75–99	370	40.1
	< 74	193	20.9
Tenure at present nursing home (years)	< 1	99	10.8
	1–2	114	12.4
	3–5	162	17.7
	6–9	168	18.3
	> 10	374	40.8
Tenure in current occupation (years)	< 1	35	3.8
	1–2	63	6.8
	3–5	126	13.8
	6–9	114	12.4
	> 10	578	63.1
Type of care units	Regular long-term	563	56.4
	Short term	101	10.1
	Palliative, rehabilitation	43	4.3
	Dementia special care	263	26.4
	Other	28	2.8
Geographic region	South-east	605	65
	Western	130	14
	Central	98	10.5
	North	98	10.5
Institution size	Small (< 40 beds)	302	32.4
	Medium (41–80 beds)	458	49.2
	Large (> 81 beds)	171	18.4

Geographic region and institution size are collected from public data

#### Response completeness

The response rates per item, mean score and standard deviation and the number of “Not applicable”, and “Item missing” are presented in Table 3. There was an overall acceptable frequency of item missing. A total of 85.5% of respondents (*N* = 796) answered all 20 items (score 1 “Never unfinished” to 4 “Often unfinished” or “Not applicable”). The highest “Item missing” (4.0%) was on Item 15: “Set up or update patients’ care plans”. The other items had 0.9–2.6% “Item missing”.

**Table 3:** Item mean scores (M), standard deviation (SD), and response distribution (*N* = 931)

			Valid responses *(N* = 794–913)				Non-valid responses (*N* = 18–137)			
							Not applicable			
Care activities in BERNCA-NH	M^a^	SD	Never	Seldom	Sometimes	Often	Activity not necessary	Not within my field of responsibility	Item missing	Total non-valid
			%	%	%	%	%	%	%	%
1. Sponge bath/partial sponge bath/skin care	1.92	0.94	40.9	28.5	21.6	5.9	0.9	1.4	0.9	3.1
2. Oral hygiene	2.09	0.96	32.4	30.6	25.8	8.1	0.3	1.5	1.3	3.1
3. Assist food/drink intake	1.82	0.93	45.4	28.6	17.2	5.6	0.8	1.2	1.3	3.2
4. Provide food other than regular meals	1.58	0.76	54.4	32.3	7.9	2.9	0.5	0.9	1.1	2.5
5. Mobilization/ change of the position	1.86	0.93	41.9	29.8	16.6	6.2	1.6	2.0	1.8	5.5
6. Leave a patient in urine/stool longer than 30 min	1.61	0.81	55.1	27.2	11.6	3.1	0.4	1.2	1.4	3.0
7. Emotional support	2.40	1.03	22.7	30.0	27.0	17.7	0.4	0.6	1.6	2.7
8. Necessary conversation with patient or family	2.04	0.92	31.8	36.6	20.4	7.7	0.8	1.1	1.6	3.4
9. Assist to the toilet when needed	1.86	0.84	39.1	36.4	18.5	3.7	0.5	1.0	0.9	2.4
10. Allow necessary time for patients to perform care themselves when possible	2.61	0.87	10.1	32.8	38.5	15.8	0.3	1.1	1.5	2.9
11. Monitoring patients as care workers felt necessary	2.29	1.00	24.7	30.9	25.9	13.3	2.0	1.5	1.6	5.2
12. Monitoring of confuse/ cognitively impaired residents & use of restraints/ sedatives	2.14	1.00	30.8	27.2	23.7	10.0	3.2	2.5	2.6	8.3
13. Keep patients waiting who rung	2.51	0.97	16.1	30.3	31.0	16.6	2.6	1.3	2.0	5.9
14. Studying care plans at the beginning of shift	2.71	1.01	13.1	27.4	28.4	26.1	1.4	1.1	2.6	5.0
15. Set up or update patients’ care plans	2.78	0.97	9.6	23.0	28.8	24.0	3.0	7.7	4.0	14.7
16. Documentation of care	2.32	0.95	22.0	34.4	29.8	11.9	0.2	0.8	1.0	1.9
17. Activity that she/he wanted	2.93	0.98	9.3	19.2	31.1	32.3	3.1	3.2	1.6	7.9
18. Experiencing community and meaning	2.54	0.94	13.9	32.7	32.9	17.0	0.6	0.9	2.1	3.7
19. Administer prescribed medication	1.82	0.80	36.6	40.9	13.4	3.4	0.3	3.2	2.0	5.6
20. Change/apply wound dressings	1.68	0.74	40.8	35.8	9.7	1.7	4.3	5.3	2.5	12.0

^a^ High scores represent unfavourable descriptions: range 1 “never” to 4 “often”

Among the NAs, 19% (*N* = 9) answered all the questions by ticking one of the response options “Never”, “Seldom”, “Sometimes” or “Often” compared to PNs: 56% (*N* = 275) and RNs: 72% (*N* = 285).

Four items (12, 15, 17, 20) had > 7% non-valid responses when the response options “Not applicable” and “Item missing” were included. Item 19 had > 3% “Not within my field of responsibility”, so this item was included for further inspection of non-valid responses. Analyses of non-valid responses, according to occupation and mother tongue of these five items show that the use of the “Not within my field of responsibility” option was highest among the NAs in item 15 (Set up and update of patient’s care plans), item 20 (Change/apply wound dressings), and item 19 (Administer prescribed medication) with 31.9, 34 and 34% NAs giving this response, respectively. Furthermore, “Item missing” was also higher among the NAs in these items. There were no statistically significant differences regarding mother tongue and use of a non-valid response, on any of the five items examined, except item 19 (Administer a prescribed medication) where “Item missing” was 5% among respondents with non-Nordic mother tongue compared to 1.5% among them with Nordic mother tongue (Additional file 1).

Concerning overall response distributions (Table 3), on item 12: “Monitoring of confuse/ cognitively impaired patients & use of restraints/ sedatives”, 5.7% of the respondents answered “Not-applicable” and 2.6% skipped the item altogether, which is high in this sample. Moreover, item 12 contains double content (monitoring and use of restraints) that could be complex to interpret and answer, and we removed item 12 prior to factor analysis.

#### Response variability

The proportion of the “Never unfinished” response, varied between 9.3% (item 17) and 55.1% (item 6). The proportion of “Often unfinished” responses ranged from 1.7% (item 20) to 32.3% (item 17). Table 3 shows the proportion of unfinished care reported in each item.

#### The subscale structure

Based on the use of “Not applicable” and the results from the interviews, three items (item 20 –Change/apply wound dressings, item 15 - Set up or update patients’ care plans, and item 19- Administer prescribed medication) is not relevant for all care worker occupations and were kept as single items. The remaining 16 items were considered appropriate for all care worker occupations and included in the scale constructing process.

697 respondents had a valid response (score 1 “Never unfinished” to 4 “Often unfinished”) on all the 16 remaining items (ratio 43:1 cases for each variable) which is adequate for undertaking explorative and confirmatory factor analysis. The assumptions for conducting a factor analysis were met [58]. There were no serious deviations from normality, and bivariate linearity between the items was confirmed. Four subscales were constructed based on theory and professional discretion. Five items were assigned to the first subscale that was labelled “routine care” and five items to the second subscale labelled “‘when required’ care”. Four items were assigned to the third subscale labelled “psychosocial care”, and finally, two items about documentation were assigned to a fourth subscale labelled “documentation”. The subscales are shown in Table 4.

**Table 4:** BERNCA-NH subscales, Mean and SD, internal consistency measures and factor loadings

	Mean (SD)	Cronbach’s α	Item-total correlations	Factor Loadings^c^
Overall scale	39.85 (21.75)	0.933		
Routine care	35.62 (23.93)	0.833		
1. Sponge bath/partial sponge bath/skin care			0.697	0.693
2. Oral hygiene			0.691	0.663
3. Assist food/drink intake			0.695	0.878
5. Mobilization/ change of the position			0.709	0.931
^a^ 10. Allow necessary time for patients to perform care themselves when possible			0.395	0.617
’When required’ care	32.27 (22.47)	0.821		
^a^ 4. Provide food other than regular meals			0.584	0.790
6. Leave a patient in urine/stool longer than 30 min			0.619	0.774
^a^ 9. Assist to the toilet when needed			0.663	0.766
11. Monitoring patients as care workers felt necessary			0.604	0.788
13. Keep residents waiting who rung			0.593	0.719
Psychosocial care	48.98 (27.08)	0.854		
7. Emotional support			0.714	0.873
8. Necessary conversation with patient or family			0.698	0.858
17. Activity that she/he wanted			0.662	0.818
^a^ 18. Experiencing community and meaning			0.706	0.841
Documentation	50.24 (28.67)	0.674		
14. Study care plans at the beginning of a shift			0.508	0.703
16. Documentation of care			0.508	0.798

CFA fit statistics: (*N* = 697) *χ*^*2*^ = 276.549, *df* = 97, *p* < 0.001, CFI: 0.996, TLI: 0.995, RMSEA: 0.052 (90% CI: 0.044–0.059), SRMR: 0.042

^a^ New/adapted question

^b^ High scores represent negative descriptions; range 0–100 for scales

^c^ Factor loadings (standardised) derived from the CFA model (N = 697)

#### Internal consistency of the subscales

In the four scales, corrected item–total correlations were all acceptable (see Table 4). All items in the scales contributed to the Cronbach’s α, except item 10 but the change was not large and the subscales´ α remained good, so we decided to keep it in the scale based on the importance of the item. Moreover, as item 10 alone is worded differently than the other items in the scale, strict adherence to α may be misguiding as similar wording of items inflate the value of α [50, 62]. The Cronbach’s α for the subscale routine care was 0.833, ‘when required’ care was 0.821, psychosocial care was 0.854 and documentation 0.674 (see Table 4). These properties indicate good internal consistency.

#### Confirmatory factor analysis

Initially, we tested different models including a one- and three-dimensional model, but fit statistics were in favour of our four-factor model. Item 1 and 2 (skin care and oral care) correlated highly (*r* = 0.789). As skin care and oral care usually are conducted simultaneously a correlation of error terms of these two items was assumed. The results showed a model with good fit to the observed data. All fit indices were within the good range, except the *χ*^*2*^ *p*-value, which were significant, which may be due to the large sample size [59]. In the final model, fit statistics were: *χ2* = 276.549, degrees of freedom (df) = 97, *p* < 0.001, CFI: 0.996, TLI: 0.995, RMSEA: 0.052, (90% CI: 0.044–0.059), SRMR: 0.042. All observed variables loaded significantly on their respective latent variable, varying from 0.62 to 0.93. The final model and factor loadings are provided in Table 4. Correlations between the four scales ranged between 0.83 and 0.90 (results in Table 5). A full version of the Norwegian BERNCA-NH can be provided upon request.

**Table 5:** Correlations (polychoric) between the four BERNCA-NH subscales in the CFA (*N* = 697)

	Routine care	‘When required’ care	Psychosocial care	Documentation
Routine care	1.000			
’When required’ care	0.906	1.000		
Psychosocial care	0.841	0.904	1.000	
Documentation	0.833	0.886	0.903	1.000

#### Validity based on relation to other variables

The hypothesized associations between the subscales and the three global ratings were supported (Table 6). The strongest bivariate correlation were with the subscale ‘when required’ care (*r* = − 0.434 to − 0.410, *p* = < 0.001), followed by psychosocial care (*r* = − 0.419 to − 0.361, *p* = < 0.001).

**Table 6:** Correlations (Pearson’s r) between scales and global rating items (*N* = 905–918)

Global rating item	Routine care	’When required’ care	Psychosocial care	Documentation
Quality of care	−0.383^a^	−0.434^a^	−0.403^a^	−0.362^a^
Job satisfaction	−0.382^a^	−0.431^a^	−0.419^a^	−0.363^a^
Recommend the unit as a workplace	−0.352^a^	−0.410^a^	−0.361^a^	−0.338^a^

^a^ Significant at the < 0.01 level (two-tailed test)

## Discussion

This study has presented a rigorous cross-cultural translation and adaptation process and evidence of the internal structure and consistency of the Norwegian version of the Basel Extent of Rationing of Nursing Care for Nursing Homes instrument (BERNCA-NH), assessed in a sample of care workers in Norwegian nursing homes.

The findings indicate that the instrument provides a valid and reliable tool with good psychometric properties to measure unfinished care in a Norwegian nursing home setting for all care workers.

In the CFA we allowed correlated error terms between item 1 and item 2. The close relationship between the items make sense in nursing home practice. Oral care is usually conducted the same time as skin care. However, it is possible only to do skin care and leave oral care unfinished. Therefore, we would not remove either of the items or collapse the items into one. In further studies of the psychometric properties of BERNCA-NH the relationship between the two items needs to be further evaluated. The four latent variables were all highly correlated. Care activities in nursing homes are related so the high correlations between the subscales in our data are not surprising. The Cronbach’s α coefficients indicate good internal consistency of the subscales. The Cronbach’s α for all the 16 items of the scale together showed good consistency. However, the α coefficient is a function of the number of items in instruments, with higher α with more items [62]. The four subscales in the Norwegian version of BERNCA-NH helps to differentiate between areas of unfinished care which also may have different implications to quality of care. The scales routine care, ‘when required’ care, documentation and psychosocial care all have an evident meaning when judging the scores. The subscales all represent dimensions of daily activity that are easily recognisable to persons familiar with long-term care in Norway. The scales provide future opportunity to monitor the occurrence of unfinished care and to study the effect of different areas of unfinished care on different outcomes. For example, if psychosocial care is left unfinished it may impact outcomes such as for example patient well-being more than unfinished documentation would.

Easy, unambiguous interpretation is of utmost importance for a measure that intends to mediate information to a potentially varied audience. It is also important that the items and the subscales are relevant for all care workers. As this was a first use of the instrument in our setting, we chose to include the response option “Not within my field of responsibility” for all items. This may have led to an all-over low “Item missing”. As some items were changed and adapted to a Norwegian context, the structure deviated from the Swiss. The four subscales were constructed based on practice experiences supported by literature [31, 56, 57]. Consequently, the instrument is country specific and cross-national comparisons must be limited to the single items that are identical.

The items that comprise the care activities which are routine care activities; predictable work at a predictable (and deferrable) time [10, 56, 57] were assigned to the first subscale labelled routine care. These care activities were the least often reported as being left unfinished, this may be because they can be postponed or left to others to perform.

The subscale ‘when required’ care comprises the care activities which have to be considered as unfinished unless they are completed promptly; The care activities in this subscale can be considered interrupting the ordinary workflow [56, 57]. When time is scarce due to for example low staffing levels, attending to ‘when required’ care may have consequences for the provision of routine care and cause further need for prioritisation.

The documentation subscale comprise the two documentation items. It is important to discern documentation from other care activities. In a previous study [18], a higher occurrence of unfinished documentation was associated with better quality of care. If documentation is left unfinished, the care workers may have the time to perform other activities that are perceived as more important to quality of care [18]. The subscale consists of only two items with a Cronbach’s α just below 0.7. Cronbach’s α is not reliable with only two items [62], but the item-total correlations were good (0.508), indicating consistency of the items in the subscale. However, the reliability of the subscale would increase adding a documentation item, preferably relevant for all care workers.

Nursing has been argued as being constrained by a “checklist” mentality, with completed practical tasks being more highly regarded than the psychosocial and interpersonal aspects of patient care [63]. Hence, as it is important to differentiate these activities, the fourth subscale was labelled psychosocial care and cover activities that tend to the patient’s psychosocial needs. Patients in Norwegian nursing homes are old, and many suffer from moderate to severe physical limitations [64], making them less capable of moving around outside. Activities outside the nursing home are also found as the least occurring regular event [65]. Input from the 14 pre-test interviews indicates that care workers did not engage in activities with patients outside the institution; instead, this was carried out by other groups of personnel only engaged in planned activities outside the nursing home. This was also a finding in the evaluation of the Swiss instrument [3]. We believe that the subscale psychosocial care in the Norwegian version consists of everyday social care activities that the patients are capable of engaging in and which care workers should practice as part of daily basic nursing care. Patients find their psychological and social need more important and meaningful than activities that maintain their physical abilities [37]. In nursing providing psychosocial care is essential [66–68]. In nursing homes, psychosocial care has a key role in optimising patient outcomes such as wellbeing, independency and healing.

Based on response patterns (Table 3) and scale means (Table 4), documentation activities are most often left unfinished, followed by psychosocial care. Unfinished care in the subscales documentation and psychosocial care are not easily noticed by colleagues and do not have immediate consequences for the patients, hence such care may also be most often left unfinished. This is in line with previous studies on unfinished care in nursing homes: the activities most often carried out and thereby prioritised the highest, are the activities the nurses expect to have immediate consequences on the patients’ health and well-being. For example, wound care and administration of medication [16, 17].

Whereas, the activities most frequently left unfinished in a hospital setting are those that are time consuming or for which it is difficult to foresee the time needed, for example, psychosocial care, planning and documenting care [10, 21, 69].

There was lower response rate among care workers with non-Nordic mother tongue. Similar results are found in studies of the general population [70–72]. There was also a low response rate among the NAs. As the group of NAs was small among the respondents, the BERNCA-NH should be further explored for relevance to this group. However, the overall small proportion of item missing indicates that the items appear relevant and easy to answer across our target population.

There were a relatively large proportion answering in the highest and lowest response category. In this study, this is probably a consequence of the 4-item response scale, with “Never unfinished” as the lowest possible and most favourable score. The same effects were found in the original BERNCA [7] and in the BERNCA-NH [3], so this is not a specific concern for the Norwegian version of the instrument. Changing the response scale with more response options (e.g. a 7-point scale) may improve the variability in the scores [73].

It could be argued that the recall period comprising the last seven work shifts may be too long, especially for part-time working respondents. However, it is essential that the activities that are listed are performed frequently in a nursing home setting. We did not test the stability of the BERNCA-NH. Due to the 7-day reference period; a test-retest was neither feasible nor relevant.

The subscale scores were associated with general ratings of care quality, work environment and willingness to recommend a unit as a workplace. Unfinished care is a quality failure per se, and the associations between the scale scores and a general rating of care quality is interpreted as supporting the BERNCA scores’ validity in this setting as studies in hospital and nursing home settings have found the same associations [7, 10, 15, 17, 18, 74]. Moreover, associations are also found with other sources of data such as urinary tract infections in nursing homes [16]. In hospital settings associations is found with mortality [14] patient falls [75] and 30-day readmission [69]. The “gold standard” for measuring unfinished care is direct observation [38]. To our knowledge, the accuracy of the evaluations of unfinished care through self-report surveys compared to this gold standard is not known. Future assessment of the instrument should explore BERNCA-NH scores compared to other quality measures such as direct observation, patient reported unfinished care, complaints, pressure ulcers, urinary tract infections, infection rates and patient falls in nursing homes.

One limitation is the low response rate in the survey. This may be the result of the web-based data collection, as some respondents reported that using a web-based survey tool was difficult. Another reason for the low response rate may be the large number of units and the geographical dispersion. We were not able to send personal reminders, but only communicate through a contact person. Therefore, we had no control of motivation and information provided to potential respondents locally in each nursing home. Surveys targeted at nurses are often characterised by low response rates, with web-based methods less successful than postal and telephone-based surveys [76]. A smaller and less dispersed nursing home sample would have made personal follow up in the units possible for the authors and this would possibly have produced higher response rates [77, 78].

The participating nursing homes were self-selected so the results cannot be generalised. However, the participating units were distributed geographically across Norway and represent different-sized facilities, traditional long-term care units and special care units for people suffering from dementia. We therefore believe that the findings can be applied to such settings.

## Conclusions

This study presents the adaptation, modification and evaluation of the Norwegian BERNCA-NH using a comprehensive method. The evaluation of the instrument provides evidence of the validity and consistency of the Norwegian BERNCA-NH assessed in a sample of care workers in Norwegian nursing homes. The instrument showed good psychometric properties and is a promising tool for measuring unfinished care in similar settings. As the instrument contains subscales, the instrument can be used to monitor different areas of unfinished care and identify areas that require improvement. The subscales differ from the original BERNCA-NH. The subscales in the Norwegian version are not comparable to the original Swiss version and cross-national comparisons must be limited to single items that are identical.

The items should be further explored for relevance in the group of NAs. Future studies on the psychometric evaluation of BERNCA-NH in nursing homes should evaluate the associations between BERNCA-NH to other measures of care such as direct observation, patient reports of unfinished care or quality indicators such as infection rates. The relationship between the items on skin care and oral care needs to be further evaluated. Furthermore, BERNCA-NH responsiveness to change and its ability to distinguish between different nursing homes needs to be evaluated.

## Supplementary information

**Additional file 1.** Non-valid responses according to mother tongue and occupation.

## Data Availability

The data are not publicly available as they form part of an ongoing PhD project at the National Institute of Public Health and the University of Oslo and will be used in further analysis and publications.

## Abbreviations

BERNCA: The Basel Extent of Rationing of Nursing Care instrument
BERNCA-NH: The Basel Extent of Rationing of Nursing Care for Nursing Homes instrument
CFA: Confirmatory factor analysis
CFI: Comparative fit index
NA: Nurse assistant
PAF: Principal axis factoring
PN: Practical nurse
RMSEA: Root-mean-square error of approximation
RN: Registered nurse
SRMR: Standardized root-mean-square residual
TLI: Tucker-Lewis index
