# Supplementary material for: Adaptation, modification, and psychometric assessment of a Norwegian version of the Basel extent of rationing of nursing care for nursing homes instrument (BERNCA-NH)
Source: BMC Health Serv Res. 2019 Dec 16;19:969. doi: 10.1186/s12913-019-4817-3 (PMC6916531; doi:10.1186/s12913-019-4817-3)
Supplement: Supplementary file 1 — Additional file 1. Non-valid responses according to mother tongue and occupation. [file 12913_2019_4817_MOESM1_ESM.docx]

**Additional File 1**.

**Non valid responses according to mother tongue and occupation (survey data) (N=931)**

**Table 1a** Percentages of “Item missing” according to occupation

|  | RN % | PN % | NA % | *p*-value ^a^ |
| --- | --- | --- | --- | --- |
| 12. Monitoring of confuse/ cognitively impaired patients & use of restraints/ sedatives | 1.5 | 3.1 | 6.4 | 0.086 |
| 15. Set up or update patients’ care plans | 2.8 | 4.1 | 12.8 | 0.004* |
| 17. Activity that she/he wanted | 1 | 2.2 | 0 | 0.235 |
| 19. Administer prescribed medication | 0.5 | 2.4 | 10.6 | <0.001* |
| 20. Change/apply wound dressings | 1.3 | 2.9 | 8.5 | 0.008* |

^a^ Pearson Chi-square * significant at the 0.05 level

**Table 1b** Percentages of “Item missing” according to mother tongue

|  | Nordic % | Non-Nordic % | *p*-value ^b^ |
| --- | --- | --- | --- |
| 12. Monitoring of confuse/ cognitively impaired patients & use of restraints/ sedatives | 2.2 | 5 | 0.075 |
| 15. Set up or update patients’ care plans | 3.7 | 5.8 | 0.245 |
| 17. Activity that she/he wanted | 1.5 | 2.2 | 0.484 |
| 19. Administer prescribed medication | 1.5 | 5 | 0.007* |
| 20. Change/apply necessary wound dressings | 2.2 | 3.6 | 0.359 |

^b^ Fisher’s exact test * significant at the 0.05 level

**Table 2a** Percentages of “Not within my field of responsibility” according to occupation

|  | RN % | PN % | NA % | *p*-value ^a^ |
| --- | --- | --- | --- | --- |
| 12. Monitoring of confuse/ cognitively impaired patients & use of restraints/ sedatives | 2.5 | 2 | 6.4 | 0.186 |
| 15. Set up or update patients’ care plans | 2 | 10 | 31.9 | <0.001* |
| 17. Activity that she/he wanted | 4.3 | 2.2 | 4.3 | 0.205 |
| 19. Administer prescribed medication | 1.5 | 1.6 | 34 | <0.001* |
| 20. Change/apply wound dressings | 2.3 | 4.9 | 34 | <0.001* |

^a^ Pearson Chi-square * significant at the 0.05 level

**Table 2b** Percentages of “Not within my field of responsibility” according to mother tongue

|  | Nordic % | Non-Nordic % | *p*-value ^b^ |
| --- | --- | --- | --- |
| 12. Monitoring of confuse/ cognitively impaired patients & use of restraints/ sedatives | 2.2 | 4.3 | 0.141 |
| 15. Set up or update patients’ care plans | 8.1 | 5.8 | 0.489 |
| 17. Activity that she/he wanted | 3.2 | 3.6 | 0.795 |
| 19. Administer prescribed medication | 3.1 | 4.3 | 0.630 |
| 20. Change/apply wound dressings | 5.6 | 3.6 | 0.414 |

^b^ Fisher’s exact test * significant at the 0.05 level

**Table 3a** Percentages of “Activity was not necessary” according to occupation

|  | RN % | PN % | NA % | *p*-value ^a^ |
| --- | --- | --- | --- | --- |
| 12. Monitoring of confuse/ cognitively impaired patients & use of restraints/ sedatives | 4.3 | 2.4 | 2.1 | 0.269 |
| 15. Set up or update patients’ care plans | 1.8 | 4.3 | 0 | 0.044* |
| 17. Activity that she/he wanted | 2.3 | 3.3 | 8.5 | 0.065 |
| 19. Administer prescribed medication | 0.3 | 0.4 | 0 | 0.851 |
| 20. Change/apply wound dressings | 4.3 | 2.4 | 2.1 | 0.269 |

^a^ Pearson Chi-square * significant at the 0.05 level

**Table 3b** Percentages of “Activity was not necessary” according to mother tongue

|  | Nordic % | Non-Nordic % | *p*-value ^b^ |
| --- | --- | --- | --- |
| 12. Monitoring of confuse/ cognitively impaired patients & use of restraints/ sedatives | 3.6 | 1.4 | 0.297 |
| 15. Set up or update patients’ care plans | 3.2 | 2.2 | 0.788 |
| 17. Activity that she/he wanted | 2.8 | 5 | 0.184 |
| 19. Administer prescribed medication | 0.4 | 0 | 0.751 |
| 20. Change/apply wound dressings | 3.6 | 1.4 | 0.297 |

^b^ Fisher’s exact test * significant at the 0.05 level
